# Coronary Flow Velocity Reserve by Echocardiography: Beyond Atherosclerotic Disease

**DOI:** 10.3390/diagnostics13020193

**Published:** 2023-01-05

**Authors:** Giovanni Civieri, Roberta Montisci, Peter L. M. Kerkhof, Sabino Iliceto, Francesco Tona

**Affiliations:** 1 Cardiology Unit, Department of Cardiac, Thoracic, Vascular Sciences and Public Health, University of Padua, 35122 Padua, Italy; 2 Clinical Cardiology, AOU Cagliari, Department of Medical Science and Public Health, University of Cagliari, 09124 Cagliari, Italy; 3 Department of Radiology and Nuclear Medicine, Amsterdam University Medical Centers, VUmc, 1081 HV Amsterdam, The Netherlands

**Keywords:** coronary flow velocity reserve, echocardiography, coronary microvascular dysfunction, coronary microcirculation, inflammation, heart transplantation

## Abstract

Coronary flow velocity reserve (CFVR) is defined as the ratio between coronary flow velocity during maximal hyperemia and coronary flow at rest. Gold-standard techniques to measure CFVR are either invasive or require radiation and are therefore inappropriate for large-scale adoption. More than 30 years ago, echocardiography was demonstrated to be a reliable tool to assess CFVR, and its field of application rapidly expanded. Although initially validated to assess the hemodynamic relevance of a coronary stenosis, CFVR by echocardiography was later used to investigate coronary microcirculation. Microvascular dysfunction was detected in many different conditions, ranging from organ transplantation to inflammatory disorders and from metabolic diseases to cardiomyopathies. Moreover, it has been proven that CFVR by echocardiography not only detects coronary microvascular involvement but is also an effective prognostic factor that allows a precise risk stratification of the patients. In this review, we will summarize the many applications of CFVR by echocardiography, focusing on the coronary involvement of systemic diseases.

## 1. Introduction

The concept of coronary flow reserve (CFR), introduced by Gould in 1974 [1,2], describes the ability of coronary flow to increase in order to match myocardial metabolic requirements. It is defined as the ratio between coronary flow during maximal hyperemia and coronary flow at rest. Compared to the resting values, this ratio may increase by up to five times during exercise and even more by administration of vasodilators [1]. The initially proposed techniques to evaluate CFR required cardiac catheterization and were therefore inadequate for their large-scale adoption. In 1991, Iliceto et al. first showed that the evaluation of coronary blood flow velocity (CFV) by transesophageal echocardiography is feasible and can be used to measure coronary flow reserve [3]. By using pulsed wave Doppler, they measured CFV in the left anterior descending artery (LAD), before and during dipyridamole infusion. Coronary flow velocity reserve (CFVR) was introduced and defined as the ratio between coronary flow velocity during hyperemic conditions and coronary flow velocity at rest. The possibility of also assessing CFVR by transthoracic Doppler echocardiography (TTDE) [4] introduced many advantages: being noninvasive, widely available, without radiation exposure, inexpensive, and easy to perform at bedside, it allowed a rapid expansion of clinical applications [5]. Although originally used mainly to assess the hemodynamic impact of an epicardial coronary stenosis [6,7], evaluation of CFVR proved useful for many other cardiac diseases. Indeed, when there are no focal stenoses of the epicardial coronary arteries, then an impaired CFVR indicates coronary microvascular dysfunction (CMD) [8]. 

**Figure 1:**
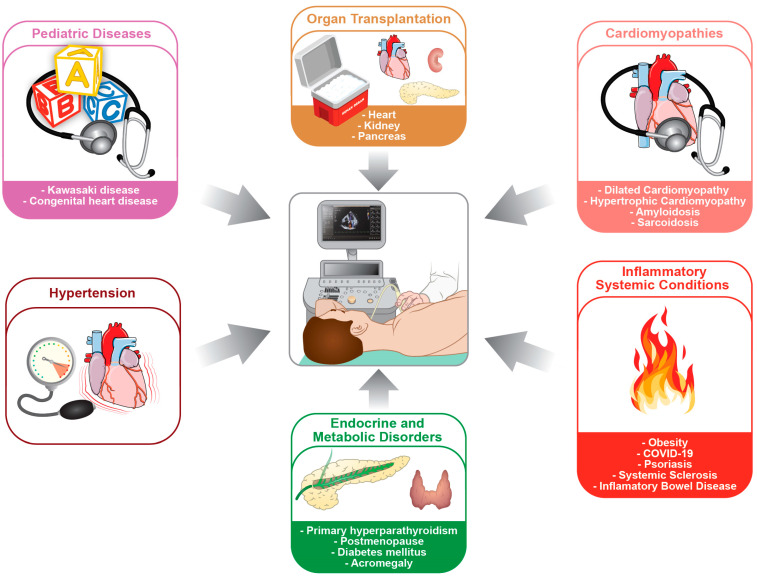
Measurement of coronary flow reserve (CFVR) by transthoracic Doppler echocardiography (TTDE) was proven to be a reliable tool with which to investigate coronary microvascular function in many cardiac and systemic diseases. Although initially developed to assess the hemodynamic relevance of an epicardial coronary stenosis, the study of CFVR by TTDE during the last 30 years expanded to a variety of different medical conditions, from organ transplantation to metabolic and inflammatory disorders and from cardiomyopathies to pediatric diseases.

In this review, we aim to summarize the applications of CFVR derived by TTDE in different cardiac and systemic diseases, in which atherosclerosis of the epicardial coronary arteries is not directly involved (Figure 1). CFVR is not only an important window on coronary microvascular function, but also an effective prognostic index that must be present in the diagnostic toolbox of modern clinicians.

## 2. Organ Transplant

### 2.1. Heart Transplantation

Cardiac allograft vasculopathy (CAV) is the major cause of mortality after heart transplantation (HT) and is a diffuse process involving the entire coronary circulation, including microvessels [9,10]. Invasive methods (such as coronary angiography, intravascular ultrasound, and CFVR by intracoronary Doppler flow wire) have for a long time been the gold standards for CAV diagnosis [11,12,13]. However, they are invasive, time consuming, expensive, and require radiation. 

In 2006, Tona et al. first used CFVR in order to improve CAV detection in heart transplant recipients. They proved that CFVR by TTDE in the LAD is lower in patients with CAV and that CFVR is negatively associated with higher CAV grades, thus being an accurate, noninvasive tool for CAV detection. Interestingly, they also found that 12% of the patients had impaired CFVR without evidence of CAV at coronary angiography, suggesting a microvascular functional impairment or a relatively low sensibility of standard angiography compared to TTDE [14]. Indeed, CFVR proved to be a reliable noninvasive marker of CAV among these patients. 

CFVR has also been used in association with wall motion analysis during dipyridamole stress echocardiography to predict the risk of CAV in HT recipients. In a group of HT patients, TTDE revealed wall motion abnormalities in only 5%, whereas CFVR was reduced in 54% of them. The combination of both served to improve sensitivity and specificity for the diagnosis of CAV [15]. Moreover, the combination of CFVR and left ventricular global longitudinal strain (LVGLS) was evaluated and provided incremental prognostic value, showing an excellent ability to rule out significant CAV [16]. 

The clinical relevance of CFVR by TTDE in HT was later confirmed by Osto et al. [17]. They demonstrated that rejection score at endomyocardial biopsies is a determinant of CFVR and is associated with CFVR reduction in patients without angiographic CAV. This supports the hypothesis that early graft vascular lesions are mostly confined to small coronary arteries and arterioles. CFVR could therefore be used to detect preclinical CAV at a stage where coronary angiography may also miss it. As proof of this, it was proven that, in these patients, the pathological substrate of CFVR reduction is represented by a hypertrophic remodeling of coronary arterioles. From a therapeutic point of view, everolimus was found to prevent the microvascular remodeling and thus the reduction of CFVR [18].

CFVR by TTDE was also analyzed as a potential marker of CAV-related major adverse cardiac events (MACEs). Lower CFVR was associated with lower MACE-free survival and a CFVR of 2.6 was identified as the optimal CFVR threshold with the best sensitivity and specificity. Furthermore, CFVR was found to be the only significant independent predictor of MACE-free survival and to be superior to standard angiography in CAV-related risk stratification [19]. Subsequent studies confirmed that CFVR can predict an event-free outcome [20], and it has been validated as a predictor of long-term outcome in HT patients without angiographic evidence of epicardial CAV. In this subgroup of patients, reduced CFVR is associated with a higher probability of new onset CAV and with a higher probability of death, regardless of CAV onset [21]. 

Recently, a complementary metric of CVFR was introduced, called companion CFVR (CCFVR) [22,23]. Its mathematical derivation is based on the notion that a dimensionless ratio (such as CFVR) is not unique, and requires consideration of a second component as represented by the companion and readily derived by applying the Pythagorean theorem [22,23,24]. Among HT recipients, it was found that various distinct microvascular patterns (endotypes) may coexist in the presence of preserved CFVR: a normal value for CFVR may therefore hamper the detection of microvasculopathy, thus preventing a correct risk stratification of these patients. However, the newly introduced CCFVR allowed a more complete characterization of the microvasculopathy and in fact enabled to the reclassification of a subgroup of patients with normal CFVR as being high risk. As a consequence of this deeper patient characterization, CCFVR was found to be a stronger predictor of mortality compared to CFVR alone [24]. In summary, CFVR, being a ratio of velocities, is dimensionless, and, therefore, not always able to capture structural CMD with low flow velocities and high resistances [22].

### 2.2. Non-Heart Transplantation

CFVR by TTDE has been used to assess the risk of cardiovascular diseases in patients who undergo non-heart solid organ transplants. 

With regard to renal transplantation, it is known that structural changes of arteries in recipients with chronic kidney disease begin many years before the onset of clinically evident CAD [25,26]. It is therefore not surprising that, in hemodialysis patients, CFVR was significantly reduced compared to controls. Moreover, 50% of the patients had impaired CFVR, implying that impairment of coronary microvasculature occurs frequently in chronic kidney disease patients [27]. It has later been shown that young recipients with low pretest probability of CAD had reduced CFVR compared to healthy controls, with a significant association with age and duration of dialysis [28]. This suggested that CFVR could identify a group of patients at high risk for cardiovascular complications but the long-term prognostic value of CFVR in kidney recipients without history of coronary artery disease has not yet been demonstrated [29].

In patients with type 1 diabetes mellitus and diabetic nephropathy who were subjected to simultaneous pancreas–kidney transplantations, CFVR could predict survival free from major cardiovascular events, being superior to standard coronary angiography in cardiovascular risk stratification. However, an inverse relationship between MACE incidence and time from transplantation was proven, suggesting a progressive reduction of cardiovascular risk after transplant [30]. As in many other cases, restorage of a physiologic systemic condition also restores coronary microvascular function.

## 3. Cardiomyopathies

### 3.1. Dilated Cardiomyopathy

CFR is impaired in patients with nonischemic dilated cardiomyopathy (DCM) [31,32]. Because patients with DCM have no epicardial stenosis that could influence coronary reserve, CFVR assessed by TTDE is related solely to microvascular state and function [33]. Santagata et al. first used transthoracic and transesophageal echocardiography to assess CFVR in these patients. They confirmed that CFVR is impaired in DCM patients compared to healthy controls and found that, at logistic multiregression analysis, the only parameter to significantly correlate with CFVR is NYHA functional class, with the lowest CFVR values found in more advanced NYHA class [34]. Impaired CFVR is due to higher baseline coronary flow velocity, reflecting higher wall stress, but also due to blunted adenosine-mediated coronary vasodilation, as reflected by the close correlation between CFVR and left ventricular end-diastolic pressure. The coronary microcirculation seems therefore subjected to compressive forces due to left ventricular dysfunction [35]. 

The prognostic role of CFVR in DCM was later investigated. Patients with DCM were followed up for a median of 22 months and, over this time, the shortest event-free survival period was observed in patients with abnormal CFVR, thus making CFVR an independent prognostic marker of bad prognosis [36]. Preserved CFVR was also found to be an independent predictor of positive response and improved left ventricular function after cardiac resynchronization therapy. This is probably due to the fact that preserved microvascular function is essential for left ventricular functional recovery [33]. However, an exhaustive pathophysiological explanation of the relation between CMD and DCM is lacking, and the clinical–histological correlation is rarely investigated [37,38]. Moreover, hemodynamic features can highly affect microvascular function and should always be taken into account [39].

### 3.2. Hypertrophic Cardiomyopathy

CFR has also been investigated in hypertrophic cardiomyopathy (HCM). In these patients, the degree of microvascular dysfunction was identified as a marker of clinical deterioration and poor survival [40] and decreased CFR is a recognized major mechanism for ischemia [41]. CFVR by TTDE was found to be reduced in HCM patients compared to healthy controls and patients with left ventricular obstruction displayed significantly lower CFVR compared to those without [42]. From a functional point of view, lower CFVR was associated with an impairment of biventricular systolic function (e.g.,: reduction of global longitudinal strain and global work efficiency). This resulted, clinically, in a significant association between lower CFVR and worse peak VO_2_ at cardiopulmonary exercise test [43], similar to the correlation of CFVR and NYHA class in DCM. Moreover, CFVR is an independent predictor of NT-pro-BNP levels [44].

With regard to prognosis, adverse cardiovascular events during follow up were more frequent among patients with reduced CFVR and impaired CFVR was an independent predictor of poor outcome even in asymptomatic patients [42]. Recurrent myocardial ischemia due to CMD might be the driver of this adverse prognostic effect. In a larger study with longer follow up, the presence of reduced CFVR enabled the identification of a subgroup of patients who had a 6.5-fold increase in the risk of adverse cardiac events. Notably, impaired CFVR appeared to be a risk marker also for all-cause mortality. These findings might indicate CFVR as an additional marker of an adverse cardiac prognosis along with the well-known clinical and echocardiographic determinants [45]. 

Analysis of different coronary arteries allowed to demonstrate that left ventricular outflow tract obstruction per se, by increasing wall stress and basal diastolic coronary flow velocities, can impair CFVR [46]. However, even vascular remodeling, reduced capillary density and compression from the outside might play a role.

### 3.3. Acquired Cardiomyopathies

Coronary microvascular dysfunction is highly prevalent in subjects with acquired cardiomyopathies, such as cardiac amyloidosis [47] and cardiac sarcoidosis [48]. CFVR measured with TTDE is significantly lower in patients with amyloidosis (both transthyretin and light chain) compared to control subjects, and a strong relation is seen between physical capacity and CFVR in this group of patients [49]. Moreover, patients with sarcoidosis displayed lower CFVR than controls, due to higher diastolic velocities at rest and lower diastolic velocities during hyperemia. However, neither the clinical severity of sarcoidosis nor the degree of systemic inflammation was related to the presence or the severity of microvascular dysfunction, and the exact cause of CFVR impairment remains unknown.

## 4. Inflammatory Systemic Conditions

The impact of chronic inflammation on coronary microvascular function is well described [50], and inflammation mediates the effects of many systemic diseases on the heart. Various conditions within this category will be addressed in detail. 

### 4.1. Obesity

Obesity, by triggering systemic inflammation, is associated with structural and functional changes in the heart [51], and the levels of inflammatory cytokines (such as interleukin-6 and tumor necrosis factor-α) are associated with long-term cardiovascular risk [52,53]. The influence of this proinflammatory state on coronary microvascular dysfunction has been investigated, and it has been shown that CFVR is significantly lower in obese patients compared to lean subjects, even after ruling out epicardial coronary stenosis. Furthermore, interleukin-6 and tumor necrosis factor-α were the only factors independently associated with CFVR. This suggests that low-grade chronic inflammation might contribute to coronary microvascular dysfunction [54]. CFVR has also been assessed in young healthy men before and after a single high-fat meal. Five hours after the high-fat meal, triglyceride levels increased significantly, and CFVR was significantly reduced [55]. This is not surprising, given the evidence that evidence that high-fat meals induce systemic inflammation [56].

### 4.2. COVID-19

Systemic inflammation is a typical feature of COVID-19 [57], and SarsCov2-associated systemic microvascular dysfunction is well described [58]. CFVR has been measured in COVID-19 patients and was found to be lower than non-COVID-19 controls. Moreover, troponin levels and the degree of CFVR reduction were significantly correlated [59]. Myocardial damage, frequent in patients hospitalized for COVID-19 [60,61,62], might then be the result of impaired coronary microcirculation [63]. CFVR impairment was also correlated to blood levels of proinflammatory markers (such as C-reactive protein and IL6) [59]. This supports the hypothesis that inflammation induced by COVID-19 causes microvascular dysfunction, which, in turn, causes myocardial ischemia and damage. 

### 4.3. Chronic Inflammatory Disease

CFR has been investigated in chronic inflammatory diseases (CIDs) such as lupus erythematosus and rheumatoid arthritis. Compared to control, CID patients had significantly lower CFVR values, and this impairment was independent of traditional risk factors for coronary atherosclerosis [61,64,65]. CFVR was further evaluated in patients with AA-amyloidosis, a long-term complication of CIDs. The AA-amyloidosis subgroup had significantly lower CFVR than the nonamyloid CID group and the presence of AA amyloidosis (together with the levels of high sensitivity C-reactive protein) could predict impairment of CFVR [66]. Similar to what has been mentioned above with regard to transthyretin and light chain amyloidosis, amyloid deposition might hinder microvascular dilation and thus impair CFVR.

Systemic inflammation might be the trigger of microvascular dysfunction in inflammatory bowel disease (IBD). Caliskan et al. recently used TTDE to measure CFVR in patients with IBD and found that it was reduced compared to healthy controls. Moreover, they found that a combined inflammatory score based on plasma albumin levels and lymphocyte count (the so called prognostic nutritional index) significantly correlated with CFVR and could independently predict impaired CFVR [67]. Interestingly, surgical resection of the diseased intestine significantly improved CFVR and the extension of CFVR improvement was greater in patients with previous CMD [68]. This finding has high clinical relevance, as it shows that microvascular function, even when markedly reduced, might recover when the pathologic noxa is removed.

### 4.4. Psoriasis

**Figure 2:**
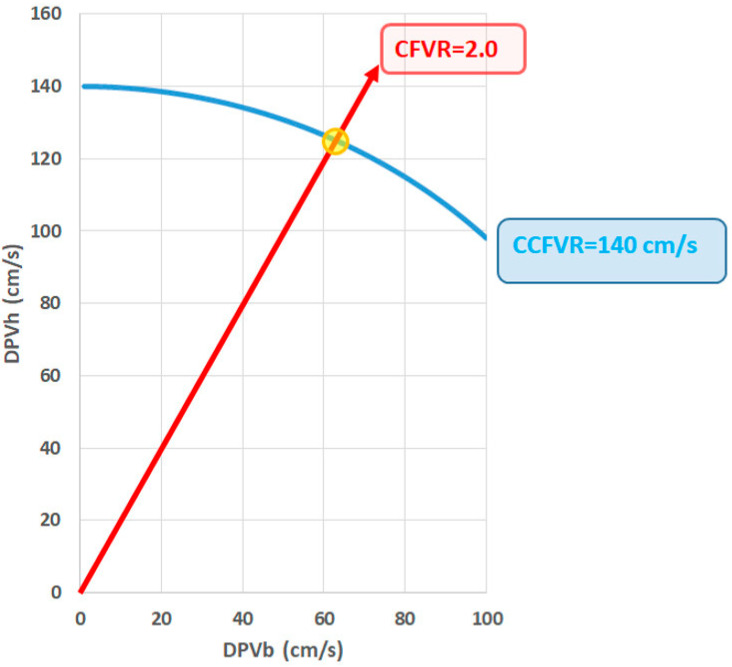
Coronary flow velocity domain representation, showing diastolic peak velocity during hyperemia (DPVh) versus diastolic peak velocity during baseline (DPVb). The traditional index refers to coronary flow velocity reserve (CFVR), defined as the ratio of DPVh and DPVb. The red line is an example of cases for which CFVR equals 2.0, and illustrates that any particular value is not unique. A second metric (indicated by the blue curve where CCFVR = 140 cm/s) is required to fully characterize the situation for an individual patient. The yellow marked point refers to the intersection of the line and the curve, corresponding with the combination DPVb = 62.61 cm/s and DPVh = 125.22 cm/s.

Psoriasis is a chronic inflammatory disease associated with an high incidence of coronary artery disease and myocardial infarction [69,70]. The effects of psoriasis on the heart, however, go beyond atherosclerotic disease and CFVR has been used to assess coronary microvascular function in these patients. In 2011, Tona et al. first reported a reduction of CFVR in patients with psoriasis without evidence of coronary artery stenosis at CT coronary angiography, showing an early impairment of coronary microvascular function. The risk of microvascular dysfunction was found to be higher in patients with a higher index of psoriasis severity, independently of conventional cardiovascular risk factors [71]. These results were later confirmed when another study proved that CFVR is decreased in patients with psoriasis and that it correlates with disease duration, disease severity and degree of systemic inflammation [72]. Moreover, in psoriasis patients, the implementation of CCFVR as mentioned above and graphically explained in Figure 2 enabled better risk stratification. CCFVR indeed could discriminate higher risk patients among those with preserved CFVR, while lower CCFVR at initial assessment could predict worse CFVR at the time of follow up [73]. 

With regard to the prognostic role, patients with reduced CFVR showed a shorter survival period free from MACEs. Screening with CFVR by TTDE might then represent a valuable, safe, and inexpensive tool for assessing the prognosis in patients with psoriasis [74]. Moreover, it is not only useful at baseline but also in the follow up during specific therapies such as treatment with TNF-α inhibitors. This therapy improved coronary microvascular function, significantly increasing CFVR in patients starting with both abnormal and normal CFVR. The observed CFVR improvement was also correlated with reduction of biomarkers of inflammation such as high-sensitivity CRP and tumor necrosis factor-α [75]. Again, CFVR can identify responders to medical therapy and allows a more precise risk stratification of these patients, especially when combined with analysis of CCFVR.

### 4.5. Systemic Sclerosis

Although clinical evidence of myocardial involvement can be found in 20–25% of systemic sclerosis (SSc) patients, at post mortem examination the heart is affected in up to 80% of them [76]. As cardiac involvement is associated with a poor prognosis [77], its early identification is of extreme clinical importance. In 2003, Montisci et al. first investigated CFVR in SSc patients and found that CFVR is significantly lower in SSc patients compared to controls. Moreover, CFVR was impaired even in the absence of clinical signs of cardiac disease [78] and epicardial coronary arteries stenosis [79], implying microcirculatory involvement. To support this interpretation, TTDE was used to assess improvement of CFVR after administration of L-propionylcarnitine (L-PC), a metabolic substance associated with beneficial effect on microcirculation. Indeed, acute administration of L-PC was associated with a short-term beneficial effect on CFVR [80]. However, microvasculopathy in SSc is systemic and the relationship between CFVR and nailfold videocapillaroscopy (NVC) abnormalities has been investigated. CFVR was inversely correlated with NVC-avascular score, confirming the systemic structural microvascular remodeling [81].

Coming to the different forms of SSc, CFVR was found to be particularly reduced in patients with the diffuse (dcSSc) type, indicating a more sever cardiac involvement in this subgroup compared to the localized one (lcSSc) [78,82]. Furthermore, this reduction appeared during earlier stages in dcSSc compared to lcSSc [83].

With regard to the prognostic role of CFVR, TTDE was used to assess coronary microvascular status in order to estimate its impact on disease outcome. Impaired CFVR was an independent predictor of death from all causes in SSc patients, suggesting that the early expression of microvascular involvement could influence prognosis independently of the cause of death [84].

### 4.6. Other Inflammatory Conditions

CFVR by TTDE has been used in a plethora of different inflammatory conditions. In burn patients, for example, CFVR is significantly lower than in controls and significantly correlates with high-sensitivity C-reactive protein and burn ratio [85]. Moreover, acute myocarditis is accompanied by alterations in coronary microcirculation. In a group of 14 patients with clinically suspected acute myocarditis, almost 60% of them showed impaired CFVR. These patients had higher levels of cardiac troponin T and larger areas of late gadolinium enhancement at cardiac magnetic resonance. Interestingly, at three-month follow-up TTDE, CFVR was normal in all patients [86]. Within the field of renal disease, TTDE has been used to assess CFVR in patients with nephrotic syndrome. CFVR was significantly reduced, and it significantly correlated with proteinuria, C-reactive protein levels, and erythrocyte sedimentation rate [87].

## 5. Endocrine and Metabolic Disorders

Untreated primary hyperparathyroidism (PHPT) is associated with increased cardiovascular morbidity and mortality; parathyroidectomy, in turn, has been shown to reduce cardiovascular risk [88,89]. Several studies found that the main reason for these findings could be the detrimental action of parathyroid hormone (PTH) on endothelial and smooth muscle cells [90,91]. The influence of PHPT on coronary microcirculation was assessed with TTDE, and it was found that CFVR was reduced in PHPT, resulting in a higher incidence of CMD in this group of patients. Moreover, CFVR inversely related to PTH levels. Interestingly, in all PHPT patients with CFVR ≤ 2.5, parathyroidectomy normalized CFVR. These results confirmed that PTH plays a crucial role in increasing the cardiovascular risk of PHPT patients [92]. 

Cardiovascular risk is also increased in acromegalic patients, mainly due to growth hormone (GH) and insulin-like growth factor (IGF)-1 induced acromegalic cardiomyopathy [93]. The influence of acromegaly on coronary microvascular function has been investigated with TTDE, and CFVR has been found to be significantly lower in acromegalic patients than controls. CFVR was inversely related to IGF-1 levels, which could, in turn, independently predict CMD. From a therapeutic point of view, treatment with somatostatin analogues could improve CFVR among acromegalic patients with CMD at baseline [94]. 

TTDE was also used to assess the relationship between menstrual cycle and CFVR and to evaluate the effect of estrogen replacement therapy in postmenopausal women. Hirata et al. measured CFVR in the menstrual and follicular phases of the same menstrual cycle and found that CFVR increased in the follicular phase compared with the menstrual phase. Interestingly, serum 17β-estradiol levels were also increased in the follicular phase. Accordingly, they found that in postmenopausal women, oral administration of conjugated estrogen increased CFVR compared to baseline. These results may contribute to explain the cardioprotective effects of estrogens [95].

### Diabetes Mellitus

Patients with diabetes mellitus (DM) are at high risk of small-vessel malfunction and disturbance of endothelium-dependent vasodilatory capacity. Moreover, microvascular involvement is a histologically well-documented complication of DM [96,97,98].

CFVR in diabetic patients with type II DM was first assessed with transesophageal-Doppler echocardiography in 1996. Patients included in the study had no clinical evidence of coronary artery disease and produced a negative stress ECG test. CFVR after dipyridamole infusion was significantly lower in patients with DM than in control subjects. Interestingly, the reduction of CFVR did not correlate with glycemic control, duration of DM and type of therapy [99]. A subsequent study revealed that CFVR was lower in DM compared to prediabetic patients, but CFVR values in the prediabetic group were not significantly lower than in the healthy controls, suggesting that microvascular dysfunction appears only after DM becomes overt [100]. The detrimental effect of DM on coronary microvascular function was also confirmed by the analysis of CFVR in patients with a history of gestational DM (GDM). CFVR values were significantly lower in GDM patients compared to women without history of GDM, and multivariate analysis showed that GDM is independently associated with CFVR reduction [101].

The evidence of CMD in diabetic patients triggered the research about potential targeted therapies, and CFVR was used to assess their efficacy. For example, it has been shown that in asymptomatic patients with type II DM, ACE inhibitors (specifically temocapril) can improve CFVR over a course of four weeks of treatment. This effect was not seen after treatment with candesartan. ACE inhibitors might therefore have more beneficial effects on coronary microvascular function compared to angiotensin II type 1 receptor antagonists [102].

With regard to risk stratification of asymptomatic DM patients, TTDE was used to assess the prognostic role of CFVR in these subgroups of patients. It was found that impaired CFVR is a strong independent predictor of adverse outcome and that it can identify high-risk patients in whom a more aggressive control of risk factors and a more frequent follow up should be advised [103]. These results were confirmed by Cortigiani et al., who analyzed the prognostic role of CFVR in patients with type II DM, chest pain (or equivalent symptoms), and evidence of either normal coronary arteries or nonobstructive CAD at coronary angiography. CFVR was again found to be a strong and independent predictor of outcome, and, interestingly, the presence of nonobstructive CAD was not associated with a worse prognosis [104]. 

## 6. Hypertension

The heart is one of the most damaged target organs in hypertension and left ventricular hypertrophy in hypertensive patients is known to impair coronary vasodilator capacity [105]. Impairment of coronary microvascular dilation, however, may also occur in the absence of left ventricular hypertrophy [106,107].

Erdogan et al. used TTDE to compare CFVR in normotensive subjects, in subjects with prehypertension and in patients with newly diagnosed and never treated hypertension. CFVR was significantly lower in the hypertension and prehypertension groups than in the control group and a significant difference was also found between hypertensive and prehypertensive patients. Moreover, the presence of prehypertension and hypertension was a significant predictor of lower CFVR [108]. This study suggested how impairment of CFVR may occur in the early phases of hypertension, before hypertrophy appears, depending on both an increase in resting coronary flow and an impairment in microvascular vasodilatation capacity [106]. Once left ventricular hypertrophy occurs, CFVR is only weakly related to LV dysfunction [109].

TTDE was also used to assess CFVR in patients with resistant hypertension, defined as blood pressure ≥ 140/90 mmHg despite treatment with three antihypertensive agents. CFVR was significantly lower in patients with resistant hypertension as compared to individuals with non-resistant hypertension, indicating a more severe impairment of coronary microvascular function that could account for the increased risk of adverse outcome in this subgroup of patients [110].

## 7. Aortic Stenosis

As stated above, CFR is known to be impaired in the presence of left ventricular hypertrophy [111,112]. In order to understand whether CFVR improves with regression of LV hypertrophy, Hildick–Smith et al. performed TTDE in patients with severe aortic stenosis, LV hypertrophy, and normal coronary arteriograms undergoing aortic valve replacement. CFVR, assessed immediately before and six weeks after aortic surgery, significantly increased after the intervention. This improvement occurred concomitantly with regression of LV hypertrophy, suggesting that this might be the main mechanism of CFVR reduction in patients with aortic stenosis [113].

The prognostic value of CFVR in patients with asymptomatic moderate or severe aortic stenosis has also been investigated. CFVR, measured with TTDE, was proven to be the strongest independent predictor of death. In particular, CFVR <1.85 had the highest sensitivity and specificity in predicting adverse outcome during follow up [114].

## 8. Pediatric Patients

Early detection and treatment of myocardial ischemia due to coronary lesions in children with Kawasaki disease (KD) is of paramount clinical importance. The first study to assess CFVR with TTDE in pediatric patients dates back to 1997. Noto et al. studied 30 patients with a history of KD and showed that noninvasive imaging of intracoronary blood flow in children, before and after adenosine infusion, could help to assess the physiologic significance of coronary microcirculation [115]. The same group also proved that values of CFVR obtained with TTDE closely correlate with those assessed invasively with Doppler guide wire, indicating that CFVR could accurately be measured by using TTDE without any invasive procedure [116]. These concepts were later further investigated and CFVR by TTDE was compared with results of single photon computed tomography (SPECT). It was found that, in children with KD, a value of CFVR <2 could predict significant epicardial coronary stenosis in the LAD and in the right coronary arteries and could predict the presence of myocardial ischemia in these territories. Moreover, CFVR by TTDE correlated with perfusion defects at SPECT [117]. With regard to the follow up, CFVR was used to prove that the endothelial function is preserved in patients with history of KD and dilated coronary artery lesions [118].

CFVR by TTDE in children has not only been used in KD, but also in congenital heart disease (CHD). Harada et al. measured CFVR in both KD and CHD patients and found that, in both groups of patients, it correlated well with CFVR obtained with Doppler guidewire examination [119]. With regard to CHD, CFVR was also assessed in patients with left ventricular volume overload due to a left-to-right shunt. CFVR was measured both in the great cardiac vein and in the LAD artery and was found to be lower in patients with ventricular septal defect than in normal children [120].

## 9. Conclusions and Future Perspectives

**Figure 3:**
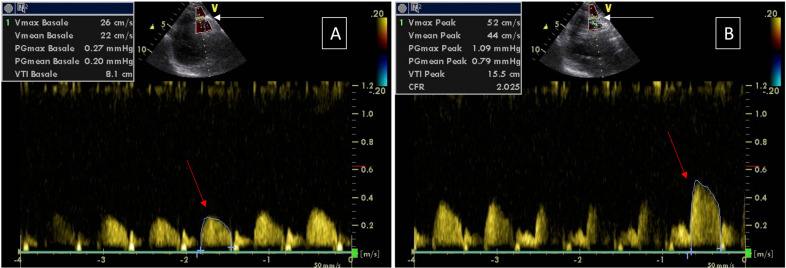
Coronary flow velocity is measured at rest left, Panel (**A**) and during maximal hyperemia right, Panel (**B**). In both conditions, pulsed Doppler echocardiography allows to measure the velocity of the blood flow in the distal part of the left anterior descending coronary artery (white arrows). To obtain coronary flow velocity reserve (CFVR), we calculate the ratio between diastolic peak velocity (cm/s) during maximal hyperemia and at rest (red arrows). In this specific patient, CFVR = 52/26 = 2.

TTDE is a noninvasive, readily available and inexpensive way to measure CFVR [5] (Figure 3). 

CFVR, in turn, can provide useful clinical information about coronary microvascular function and correctly stratify the cardiovascular risk of many different groups of patients. From its first validation in 1991 [3], the field of application of CFVR has rapidly expanded, ranging from quantification of coronary stenosis to the prognostic prediction in cardiomyopathies and inflammatory diseases. However, despite its multiple areas of clinical application, CFVR measurement by TTDE has not become a routine diagnostic test, and only few experienced centers use it in their daily clinical practice. From a technical point of view, the main obstacle to its widespread utilization might be the necessary sonographic skills, which require a steep learning curve that may discourage clinicians. However, once familiar with the technique, technical feasibility is high (up to 90% [121]) and intra- and interobserver variability is low [122] (5%), even among obese patients [54]. From a mathematical point of view, CFVR is a dimensionless ratio and therefore not ideal to explore differences between groups, as same direction changes in numerator and denominator may readily cancel out. A logical, mathematically derived companion (CCFVR, as cited above) complements CFVR [23,24,73], enabling patient characterization superior to that achieved by CFVR alone. Once becoming more popular among clinicians, the additional value of CCFVR might then be the trigger to a more widespread investigation of microvascular function by TTDE. Indeed, CCFVR can be calculated from the same data required for CFVR and no further procedures are required. 

CFVR by echocardiography, despite being introduced more than 30 years ago, is still a vibrant area of research with heterogeneous clinical perspectives. More comprehensive mathematical analysis and a deeper knowledge of its applications will expand the utilization of echocardiography to investigate coronary microvascular function in the near future. 

## Data Availability

Not applicable.
